# Evaluation of aspiration risk by relatives of inpatients in the neurology service: A metaphor analysis

**DOI:** 10.1111/hex.13883

**Published:** 2023-10-02

**Authors:** Canan Kaş, Filiz Ö. Çakır, İdris Kocatürk

**Affiliations:** ^1^ Department of Midwifery Health Sciences Faculty, Kastamonu University Kastamonu Turkey; ^2^ Department of Nursing Health Sciences Faculty, Kastamonu University Kastamonu Turkey; ^3^ Department of Neurology Kastamonu Training and Research Hospital Kastamonu Turkey

**Keywords:** fear, metaphor, qualitative research, respiratory aspiration, risk

## Abstract

**Background:**

The risk of aspiration is high in stroke patients due to dysphagia/loss of swallowing. This problem can cause problems affecting the nutrition of patients. Due to the possible risk of aspiration during feeding, patient relatives are hesitant to feed their patients. Because of this fear, malnutrition and hospital readmissions may increase. It is important to evaluate the fear of aspiration risk of relatives of patients hospitalized in the neurology service. The aim of this study is to determine the views of the relatives of patients treated in the neurology service about the risk of aspiration through metaphors.

**Method:**

This article analyzed metaphors of patients' relatives' about the risk of aspiration. The analysis uses metaphor identification and analysis. The research sample consisted of 31 patients. First of all, in the study, evaluation of the data was conducted by performing content analysis, as a result of which it was divided into metaphors and conceptual categories, and the relevant field was associated with the text (event, thought, feelings, related in speech or writing). In the reporting of research, the Consolidated Criteria for Reporting Qualitative Research (COREQ) checklist was used.

**Results:**

It was seen that the patients' relatives produced 31 different metaphors in response to the statement ‘Aspiration risk is like …, because it is …’ The patients' relatives mostly compared the concept of ‘fear of aspiration’ to the concept of ‘fear’ (*n*: 24).

**Conclusions:**

In this study, relatives mostly described the concept of fear related to the risk of aspiration. According to this result, patients' relatives have a great fear of aspiration increasing the risk of readmission to hospital and of malnutrition. This result shows that informing patients' relatives will increase awareness and will allow them to provide informed care.

**Patient and Public Contribution:**

Collaborated with patients and their relatives regarding the risk of aspiration and contributed to the planning of care for the risk of aspiration.

## 1 INTRODUCTION

Aspiration is the unintended entry into the respiratory tract of material being sent to the stomach while swallowing.^1^ Patients with neurological disorders may lose the ability to eat and drink because of consciousness disorders, swallowing problems, posture disorders, reduced mobility, communication problems, fatigue, depression or visual perception disorders,^2^ as well as damage to the central or peripheral nervous system, dysphagia deriving from muscular and neuromuscular junctions.^3^ Complications of dysphagia include inadequate nutrition, dehydration, weight loss, and respiratory tract infection.^4^ It leads to respiratory tract obstruction, hypoxia and aspiration pneumonia.^5^ Because of the aspiration risk, there is a high risk of the development of malnutrition in patients. Catabolism is activated by malnutrition, and this is among the factors increasing the risk of pneumonia.^6^ Assessment of dysphagia is important to reduce the risk of aspiration. Dysphagia assessment procedures are selected according to patient characteristics, the seriousness of the swallowing disorder, and the appropriateness of the procedure.^7^, ^8^ Assessment of aspiration risk and informing the patient's relatives about this are important. Education of patients' relatives is among the procedures that reduce the anxiety of patients and their relatives and increase people's adaptation to the disease, which form part of nursing practices. In the study of Robinson et al.^9^ in which family carers experience dysphagia after a stroke, and Davis et al.^10^ in their study of contributors to poststroke dysphagia‐related caregiver burden, attention was drawn to the importance of dysphagia and the risk of malnutrition due to the risk of aspiration. However, the emotional state of caregivers about the risk of aspiration was not addressed.

Metaphor is an indicator of an individual's perceptions regarding a concept, and a way of expressing positive or negative emotions through their own ideas.^11^ Metaphors carry specific meanings and are a means of contributing to the formation of a point of view.^12^, ^13^ At the same time, metaphors allow a person's previous experiences to be directed from one type of understanding to another through the mind and cause the formation of different structures regarding the concepts.^14^ Determining a metaphoric interpretation to understand the relationship of patients' relatives with aspiration is of value to gain a different understanding for the nursing view. Determining patients' relatives' aspiration and reducing patients' time in the hospital by giving education on this is important for preventing readmission and providing continuity of care. In this regard, it is thought that the metaphoric perceptions of patients' relatives will bring a new dimension to nursing care. In this study, it was planned to determine the views of patient relatives about aspiration through metaphors.

### 1.1 Research aim

The aim of this study was to determine by means of metaphors the views of the risk of aspiration of the relatives of patients admitted to the neurology service.

It is thought that the results of this research will show the views of aspiration risk, and thereby reduce patients' readmission to hospital and contribute to the provision of education to remove the risk of malnutrition.

Answers to the following questions were sought in this research:

1. What are the metaphors which the relatives of patients in the inpatients' neurology service make for the concept of aspiration?
2. Which conceptual categories can these metaphors be gathered under?

## 2 METHODS

### 2.1 Design

The research was conducted at neurology inpatient services nos. 1 and 2 of a teaching and research hospital in the Western Black Sea region of Turkey.

Phenomenology, a quality research design, was used in the research. The phenomenology design focuses on phenomena of which we are aware but which we do not have a deep or detailed understanding of. Phenomenology is a method assessing lived experience. In phenomenology, to discover the meanings underlying the phenomenon, it is first of all experienced by individuals. Phenomenology attempts to describe the world and explain the essence of lived experiences. It is emphasized that there is a connection between the person experiencing the phenomenon and the phenomenon itself, and the phenomena themselves constitute the starting point of phenomenology.

The phenomenology pattern is a research design that comes from philosophy and psychology. In this research, the researcher tries to analyze the common experiences of individuals about a phenomenon. In this study, which was conducted with the experiences of a small number of people, the researcher reaches the essence he wants to reach in the research thanks to the data he obtained.^15^, ^16^ The purpose of the phenomenology pattern is to reveal how people define objects and behaviours through categories.^17^ Hermeneutic phenomenology is used in metaphor studies. Interpretive phenomenology (hermeneutic phenomenology) was interested in the life world and focused on human experiences.^18^ The researcher examines the interpretation of ‘life texts’ experienced in interpretive phenomenology and the phenomenon he deals with. In this process, the researcher identifies the main themes that constitute the nature of lived experience. The researcher seeks the answer to the question of what, what, and how causes people to have this experience. The researcher, who carries out his research with hermeneutic phenomenology, begins to write a description of the phenomenon he is dealing with after identifying his themes. The most important feature of hermeneutic phenomenology research is that it has a research process based on both the description of the phenomenon and its interpretation. In other words, in this research, the researcher reveals what the lived experiences of individuals mean and comments on them.^19^ Hermeneutic phenomenology allows both interpretation and description in research. Interpretive phenomenology explores the history, background, culture and social environment behind experiences by uncovering details. Therefore, different meanings derived from the common experiences of the participants need to be interpreted.^18^

This research method borrows experiences from individuals to describe and interpret them. All phenomena experienced by individuals can be researched and explained. Through phenomenology, an attempt is made to reveal the experiences and perceptions of a phenomenon and the meanings attached to it.^20^, ^21^

When determining the target area in metaphor analysis, first the topic is decided on, then the questions to be asked are decided, and a template is made of the assessment process.^22^ After these preparations are completed, the metaphors are collected in relation to the research and later, by grouping, the topic headings are formed. It is of vital importance that the researchers understand and correctly analyze the metaphors used in order for the research to be conducted correctly. It is necessary to assess in detail the analysis which is to be performed within the topic headings prepared.^23^, ^24^

The research sample in this study consisted of the relatives of inpatients in the neurology service of a teaching and research hospital. Sampling was performed directly, and the sample consisted of the relatives of patients at risk of aspiration who consented to participate in the study. A total of three patients' relatives did not consent to participate in the study between 15 August and 30 December 2022, when data were collected, and a final total of 31 relatives of patients at risk of aspiration made up the sample. The metaphors produced by the patients' relatives were identified as concepts, and a list was created. In this way, care was taken as to whether the metaphors were stated clearly.

### 2.2 Data collection

A sociodemographic characteristics question form and a metaphoric perceptions data collection form were used as data collection instruments.

*Patient description form*: This form was prepared by a researcher according to information in the literature, and consisted of five questions concerning the patient's age, gender, education level, relationship to the patient and patient caregiving time.

*Metaphoric perceptions data collection form*: This form was created by the researchers. It contained the sentence ‘Aspiration risk is like …, because it is …. ’ The relation between the topic and source of the metaphor was examined with the word ‘like’, and the meaning and reason loaded on the metaphor were brought out by the world ‘because’.

Data collection was performed by face‐to‐face interview, completing the above‐mentioned forms. The patients' relatives were asked to compare the phenomenon of aspiration risk with something else—a living being, and object or anything‐and to state in a short way the reason for this comparison. This took approximately 10 min. Data collection was performed by the data collection researchers by asking the patients’ relatives the questions on the form.

### 2.3 Data evaluation

Content analysis is performed to establish themes that can be easily understood by someone reading raw data acquired either orally or in writing.^25^ According to Holsti, content analysis is a research technique that allows inferences to be made through the objective and systematic description of salient features of messages.^26^ Stages of content analysis.

The stages of content analysis are described in six items.

1. Defining the research problem.
2. Determining the research universe and sampling selection.
3. Creating research categories and describing.
4. Creating the coding ruler.
5. Testing and reliability of the coding scale measuring.
6. Data entry. Analysis and interpretation.^27^

First, in the study, the data were examined with content analysis. Second, the *metaphors* determined as a result of content analysis were separated into *conceptual categories* and the relevant field was associated with the thoughts in the metaphor. In order for the patients' relatives to show their concept perceptions of the risk of aspiration, it was stated as metaphors ‘(because it is “…….”)’ Categories were formed according to the integrity of the meaning. In the analysis of data, frequency values (*f*) were also examined.

The analysis and interpretation of data is performed in five steps.^28^ In this study, the analysis of data was performed in the five stages listed below using the content analysis technique.

1. *The coding and sorting stage*: First of all, the data was sorted. Each complete item of data was assigned a code number. The code ‘A1’ formed the code of the metaphor produced for the aspiration. A drawing of the metaphor image was created according to the answers given by the participants.
2. *The sample metaphor compilation stage*: The metaphors produced by the participants were given codes and analyzed. The relation between similar metaphors produced was examined.
3. *The stage of forming categories*: The metaphor images created by the participants were grouped by their similar characteristics relating to the concept of aspiration. Three valid aspiration risk metaphors formed by 31 patients' relatives were grouped into conceptual categories. Each metaphor was associated with the meaning and perception loaded onto the concept of aspiration by the patients' relatives.
4. *The validity and reliability stage*: The number of patients' relatives participating in the research, and in the categories and sub‐headings obtained as main themes from the concepts, the approximate proportion of metaphors with the power to represent the conceptual categories were determined. To ensure reliability, after the categories were formed, two teaching staff members were given a paper on which the metaphors and criteria were written and the names of the conceptual categories were written, and they were asked to complete it. All of the metaphors were matched with a category. Then, the teachers' matching of the metaphors and categories formed were compared with the researchers' matching. The result of this matching was applied to the formula reliability = unity of view/unity of view + difference in view.^29^ The reliability of the metaphor categories was calculated as in this formula. As a result, reliability was calculated with the formula 2/2 = 1. The result obtained was interpreted as showing that the study was reliable.
5. *Transfer of data to computer:* The metaphors were separated into categories according to the criteria, and the frequency (*f*) of participants in the categories which they represented were calculated. The metaphors were separated into categories according to the criteria, coded according to participants, and coded. The metaphors emerging as a result of the research were categorized taking account of their common characteristics and similarities. In the analysis of the descriptive characteristics of the patients' relatives, the programme Statistical Programme for the Social Sciences 21.0 was used, and frequency and percentage values were calculated.

## 3 RESULTS

Information was given about participants' descriptive characteristics and the metaphors which they developed for the concept of aspiration. The mean age of the participants was 52.58 ± 2.287 (minimum: 24, maximum: 76) years, 64.5% were female, and 67.7% had been caring after a patient for less than 3 months. Also, 58.1% of the patients who they were cared for after had not previously experienced the risk of aspiration (Table 1).

**Table 1:** Descriptive characteristics of patients' relatives (*n* = 31).

Characteristics	Frequency (*f*)	Percentage (%)
Age *X̄* ± SS (min–max) 52.58 ± 2.287 (min: 24, max: 76)		
Gender		
Female	20	64.5
Male	11	35.5
Education level		
Primary school	1	3.2
Secondary school	13	41.9
University	11	35.5
Postgraduate	6	19.4
Relationship		
Spouse	10	32.3
Child	15	48.4
Parent	1	3.2
Relative	4	12.9
Caregiver	1	3.2
Duration of caregiving		
Less than 3 months	21	67.7
3–6 months	5	16.1
More than 12 months	5	16.1
Patient's previous risk of aspiration		
Yes	13	41.9
No	18	58.1

Abbreviations: max, maximum; min, minimum.

Table 2 shows the 31 metaphors produced in response to the statement ‘Aspiration risk is like …, because it is …’ Most of the participants compared aspiration risk to fear (*f*: 24, 77.4%), trepidation (*f*: 1, 3.2%), a fine line (*f*: 1, 3.2%), lack of control (*f*: 1, 3.2%), death (*f*: 3, 9.7%) or vomiting (*f*: 1, 3.2%).

**Table 2:** Metaphors produced by the patients' relatives for aspiration risk.

Metaphor code (MK)	Metaphors	Frequency	%	Participants' statements on reasons for metaphors	*n*	%
1	Fear ‘Aspiration risk is like fear, because it is …’	24	77.4	Death	12	50
				The end	1	4.16
				Congestion	10	41.66
				Continuous	1	4.16
2	Trepidation ‘Aspiration risk is like trepidation, because it is …’	1	3.2	Fear	1	3.2
3	A fine line ‘Aspiration risk is like a fine line, because it is life or …’	1	3.2	Life and death	1	3.2
4	Lack of control ‘Aspiration risk is like lack of control, because it is …’	1	3.2	Involuntary	1	3.2
5	Death ‘Aspiration risk is like death, because it is ….’	3	9.7	Fear	1	33.34
				Obstruction	2	66.66
6	Vomiting ‘Aspiration risk is like vomiting, because it is …’	1	3.2	An inability to swallow	1	3.2

**Figure 1:**
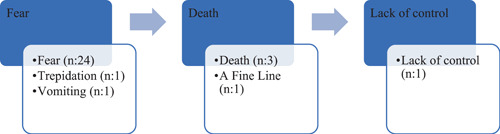
Categories of metaphors obtained by content analysis.

The categories of metaphors obtained as a result of content analysis are presented visually below in Figure 1. It is seen that there are three metaphors: fear, death and lack of control (Figure 1). According to this result, the patients' relatives had similar perceptions, and gave metaphor perceptions which would increase the risk of malnutrition.

## 4 DISCUSSION

Metaphors are a powerful cognitive means of understanding and explaining an abstract, complex or hypothetical phenomenon. Metaphor is not only a figure of speech but also a figure of thinking.^30^ The brain turns one concept into another concept, and functions by the method of making concrete something which is abstract at the level of perception. The aim of this study was to set out the metaphor perceptions of the aspiration risk of the relatives of patients of neurology inpatients with a risk of aspiration. The metaphors on the aspiration risk produced by the patients' relatives were categorized as fear, death, lack of control, trepidation, a fine line and vomiting.

### 4.1 Fear

Aspiration is a frightening symptom because it occurs suddenly and it has a negative effect on those close by. It is known that a positive or negative attitude to a state or phenomenon shapes a person's behaviour in the same way, and the attitude to aspiration fear is important in that it affects behaviour in caring for the patient. Aspiration because of dysphagia in stroke patients increases the risk of pneumonia, and is an important risk factor in increasing the symptoms of fear and inadequate nutrition of the patient.^31^, ^32^ It has been reported that fear and anxiety levels are increased in both patients and their caregivers. The reasons for fear and anxiety in caregivers have been reported as the sudden increase in caregiving responsibility, feelings of being prepared for the worst and changing role responsibilities, and in the patients being cared for, coughing or suffocation and inadequate nutrition.^33^, ^34^ In another study, it was reported that caregivers experienced fear and stress regarding their inability to meet the patient's nutritional needs and the possibility that the patient would suffocate.^35^ Dysphagia in older adults opens the way to worry in caregivers over the fear of suffocation, concerns about nutrition and caregiving responsibilities.^36^ Caregivers of those with head and neck cancers or stroke and other neurological problems reported an increasing fear of dysphagia, worry, sadness, guilt and loneliness, decreasing support and social participation outside the home, and in general a decreased quality of life.^37^ Bushuven et al.^38^ found in a study with the caregivers of patients with dysphagia that the patients experienced more frequent sadness and anger than the health service providers, and that family members more often felt worry. In a qualitative analysis, 20 themes emerged: seven anger, two pleasure, four sadness, three anxiety, two disgust, one shame and one punishment. In the same study, patients mostly spoke of the fear of suffocation, and of the fear of professionals making a mistake.^38^ In the present study, aspiration fear was most often associated with the concept of fear. This result indicates that patients' relatives feel excessive fear of aspiration and that the risk of readmission to hospital and malnutrition are increased. In line with this result, it can be said that informing patients' relatives will increase awareness, and taking patients into informed care will prevent readmissions.

### 4.2 Death

Any delay in diagnosis or treatment generally results in high mortality.^39^ Dysphagia after a stroke is a frequently seen complication of acute stroke, and is related to increased mortality, morbidity and hospitalization due to aspiration pneumonia and malnutrition.^32^ The death rate for pneumonia associated with aspiration in stroke patients is approximately 35%.^40^ One of the metaphors produced in the present study was the metaphor of death. When stroke patients compare the aspiration with death, early detection of dysphagia and training to patients' relatives must be provided. Improving patients' quality of life by reducing to a minimum the probability of aspiration pneumonia is a nursing duty.

### 4.3 Lack of control

Lack of control is a term used for suddenly developing events that can't be controlled. In the statement of the patients' relatives ‘Aspiration risk is like lack of control, because it is involuntary’, aspiration was compared to lack of control because it was thought of as being unpreventable. When caring for patients at risk of aspiration, the main target is prevention. Because aspiration in stroke patients frequently results in pneumonia, scanning for dysphagia can significantly reduce the risk of aspiration pneumonia, and this will remove the lack of control.^41^

### 4.4 Trepidation

Trepidation is defined as ‘fear or worry about what is going to happen’ or ‘painful agitation in the presence or anticipation of danger’.^42^ Stroke causes a wide range of neurological problems that affect eating. Therefore, dysphagia is reported in the literature to be of special concern due to the potential for airway obstruction, malnutrition, and aspiration pneumonia.^43^ In particular, caregivers of older adults with dysphagia may experience fear of choking, feeding concerns, distress at the prospect of changing feeding behaviours or feeding tubes, and anxiety over caregiving responsibilities. Caregivers of older adults living in the community have also reported increased fears and concerns about nutritional status and choking.^44^ In this study, it was thought that the relatives of the patients experienced this feeling because they had not cared for a patient in this way before and had not received training on this subject.

### 4.5 A fine line

A fine line is defined as ‘a very small difference between two things that may seem different’.^45^ Here, caregivers described the risk of aspiration as a fine line between life and death. Dysphagia has serious consequences, such as choking and aspiration pneumonia, which can be fatal.^46^ Aspiration pneumonia is one of the most common causes of mortality after stroke.^47^ Therefore, it was thought that caregivers of patients at risk of aspiration saw this as a situation that could pose a danger to the patient.

### 4.6 Vomiting

Vomiting is defined as ‘the act of emptying the contents of the stomach through the mouth’.^48^ Patients with dysphagia spend more time and effort moving food from the mouth to their stomach, increasing the risk of aspiration of oropharyngeal contents.^49^, ^50^ Among the different aspirations, food aspiration is common in debilitated patients such as stroke patients, leading to a high risk of aspiration pneumonia in these patients.^51^ Uncontrolled nausea and vomiting may adversely affect treatment compliance, interfere with enteral absorption of drugs, reduce the quality of life and cause complications such as dehydration, nutritional deficiencies, electrolyte imbalance and aspiration pneumonia.^52^, ^53^ Aspiration pneumonia is one of the most important complications among hospitalized stroke patients.^54^ Lidetu et al.^55^ found that the risk of developing aspiration pneumonia in patients with vomiting and dysphagia was higher than in patients without vomiting and dysphagia. For these reasons, it is considered natural for caregivers to associate nausea and vomiting with aspiration. In the literature, Rangira et al.,^56^ it was found that fear of aspiration is among the causes of care burden in caregivers of patients with dysphagia. The inability to swallow may develop as a consequence of complications of stroke. As a result, it is thought that the patient's relatives are afraid of the development of vomiting and the complications that may occur as a result of vomiting.

### 4.7 Strengths and limitations

Because the patients' relatives who participated in the study used their own statements, they were not affected by each other, and this increases the veracity of the data.

The study was conducted at a single centre, in a teaching and research hospital in Turkey. The findings obtained from the research represent the views of the relatives of 31 patients hospitalized in the neurology service of the hospital, who were at risk of aspiration. For this reason, the findings cannot be generalized to all patients' relatives.

## 5 CONCLUSION

In our study, the aspiration risk was most frequently defined together with the concept of fear. This result shows that patients' relatives have a great fear of the risk of aspiration and that the risk of readmission to hospital and malnutrition are increased. In addition, there may be a possibility of neglecting the patient's nutrition due to the fear experienced by the patient's relatives. It shows that informing patients' relatives will increase awareness and that this will provide patients with informed care. The metaphors mentioned in the study may also negatively affect patient care. Therefore, it may be recommended to increase research on this subject.

## AUTHOR CONTRIBUTIONS

Canan Kaş was involved in the study conception, designed data analysis, wrote the first draft of the manuscript, and approved the final version to be published. Filiz Ö. Çakır participated in the study conception, designed data analysis, and approved the final version to be published. İdris Kocatürk was involved in the study conception and approved the final version to be published.

## CONFLICT OF INTEREST STATEMENT

The authors declare no conflict of interest.

## ETHICS STATEMENT

Written approval (Decision No. 2022/1009, dated 4 July 2022) to perform the study was obtained from the Clinical Research Committee. Institutional permission was obtained to collect the data. The participants were given information on the research, and their informed voluntary consent was obtained. All directives of the Helsinki Declaration were followed and informed consent was obtained from the participants.

## Data Availability

The data that support the findings of this study are available on request from the corresponding author. The data are not publicly available due to privacy or ethical restrictions.
